# Dr. Adolph Petermann

**Published:** 1879-05

**Authors:** 


*Dr. Adolph Petermann,* the author of the European work entitled the "Dental Almanac," a notice of which appears as a Bibliographical Notice in the present number of this Journal, deserves and should have all possible encouragement which the Dental Profession in this country can give him, for his successful efforts in checking the swindle perpetrated by certain parties in the sale of Medical and Dental Diplomas from bogus institutions. The position which Dr. Petermann has taken on this question, and his untiring efforts to protect the communities throughout which these worthless documents have been distributed, have subjected him to the combined attacks, not only of the parties engaged in this nefarious business as principals, but also to those of the persons who have become the victims, or accomplices of the leaders of the movement. These attacks have also been directed against the old and respectable Dental Colleges of this country; and the parties making them are endeavoring to show, on the other hand, that the institutions they profess to represent (under high sounding names) are *bona-fide* establishments, Public and State Institutions, Universities, as understood in Germany, etc. In fact Dr. Petermann has almost made a martyr of himself. His efforts have never been properly appreciated, indeed, hardly known in this country. We trust that our dental journals will notice the commendable efforts of Dr. Petermann, so that the profession may properly appreciate his good work.

